# Spinal Dissemination of Pineal Parenchymal Tumors of Intermediate Differentiation Over 10 Years After Initial Treatment: A Case Report

**DOI:** 10.7759/cureus.57147

**Published:** 2024-03-28

**Authors:** Airi Miyazaki, Keishi Makino, Naoki Shinojima, Shinji Yamashita, Yoshiki Mikami, Akitake Mukasa

**Affiliations:** 1 Neurosurgery, Kumamoto University Hospital, Kumamoto, JPN; 2 Neurosurgery, Kumamoto City Hospital, Japan, JPN; 3 Neurosurgery, Miyazaki University Hospital, Miyazaki, JPN; 4 Pathology, Kumamoto University Hospital, Kumamoto, JPN

**Keywords:** long term survival, radiotherapy (rt), kbtbd4 mutation, spinal dissemination, pineal parenchymal tumor of intermediate differentiation (pptid)

## Abstract

Pineal parenchymal tumors (PPTs) are rare, accounting for less than 0.3% of all primary central nervous system (CNS) tumors. Pineal parenchymal tumors of intermediate differentiation (PPTID) (WHO grade 2 or 3) show an intermediate prognosis between pineocytoma and pineoblastoma. The clinical course is unknown, and the optimal treatment for PPTID, especially for recurrence, has not been determined. We report a case of PPTID with spinal dissemination over 10 years after treatment and survival for four years. A 56-year-old woman presented with headaches and diplopia. Computerized tomography (CT) and magnetic resonance imaging (MRI) revealed a pineal mass, but leptomeningeal dissemination was not identified on whole-spine MRI. Microsurgical gross total tumor resection (GTR) was performed, and the pathological diagnosis was PPTID (grade 3). In addition, a later study found it to harbor a *KBTBD4* mutation. She underwent whole-brain radiation therapy with a focal boost. The patient was unable to continue chemotherapy for severe myelosuppression after the first course of treatment. Eleven years after the surgery, she was unable to walk, and a whole-spine MRI revealed multiple masses at C3-4, T4, and cauda equina. Fluorodeoxyglucose-positron emission tomography (FDG-PET) revealed accumulations of the same lesions. No recurrence was observed in the brain. A biopsy of the caudal portion was performed, and the histopathological findings were the same as those of the initial surgery. Spinal dissemination was refractory to chemotherapy but responded to whole spine radiotherapy with focal boost, and she remained tumor-free for four years. We considered good local control with a combination of GTR and subsequent radiation therapy to contribute to long-term survival. The timing of spinal radiation administration is controversial because of the tendency for late cerebrospinal dissemination. The importance of long-term follow-up of the spine and head is emphasized. In PPTID cases with good local control, withholding spinal radiation until spinal dissemination occurs may become a long-term treatment plan.

## Introduction

Pineal parenchymal tumors (PPTs) are rare, accounting for less than 0.3% of all primary central nervous system (CNS) tumors [1]. The WHO classification of CNS tumors (2021 revision) subdivided pineal tumors into four grades: pineocytoma (WHO grade 1), pineoblastoma (WHO grade 4), and pineal parenchymal tumors of intermediate differentiation (PPTID) (WHO grade 2 or 3). It was first classified by the WHO in 2000 as a pineal parenchymal tumor with an intermediate prognosis between pineocytoma and pineoblastoma. The determination of the mitotic index and immunohistochemistry are used to pathologically classify PPTID as grade 2 or 3 [2,3]. PPTID may account for up to 45% of pineal tumors [4]. There is a slight female predominance, and they are more common in middle-aged patients [5]. Unlike other histological subtypes, the optimal management for PPTID remains to be determined due to its relatively recent characterization and rarity. The reported five-year survival rates of these tumors are 74% for WHO grade 2 PPTID and 39% for WHO grade 3 PPTID [6].

In this study, we report a case of PPTID with spinal dissemination over 10 years after treatment and survival for 4 years.

## Case presentation

**Figure 1:**
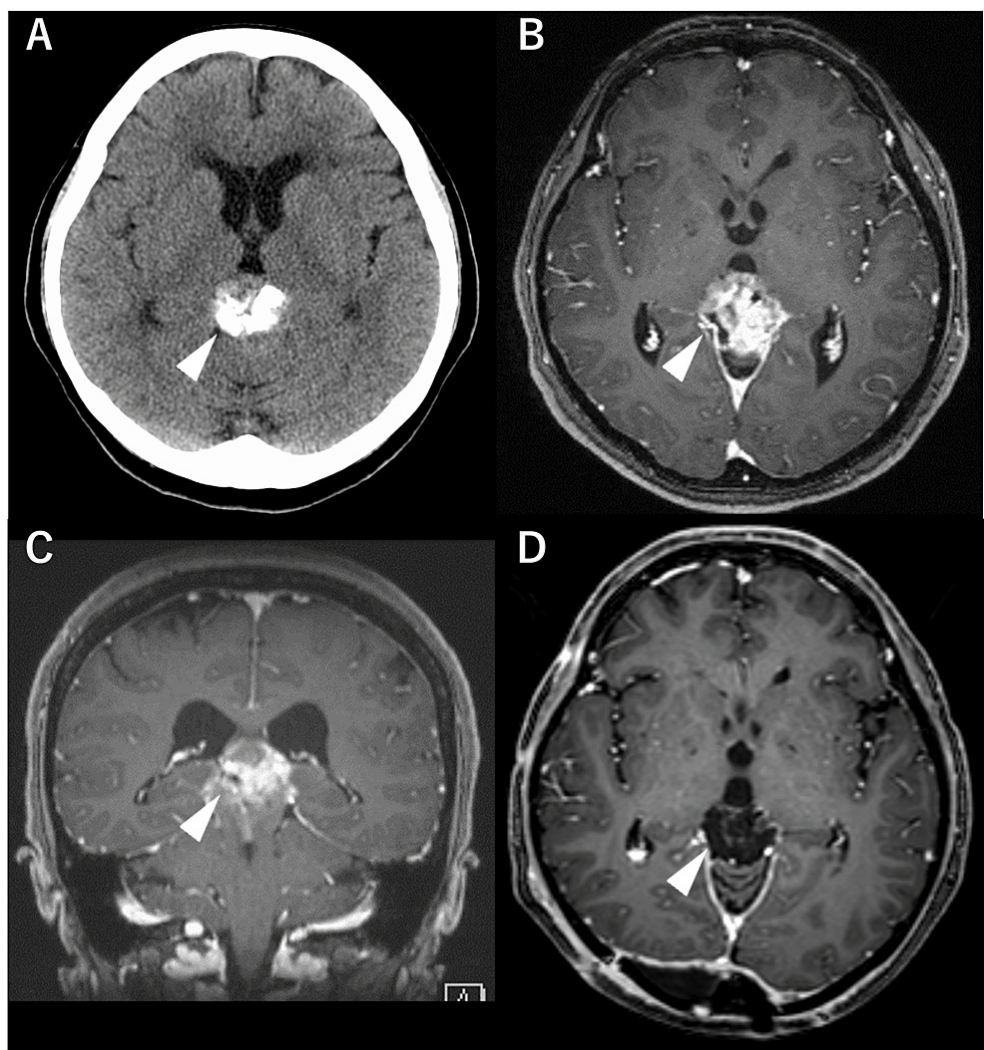
Preoperative and postoperative images Preoperative CT (A) shows a mass lesion with calcification in the pineal region (arrowhead). The mass is well enhanced on axial (B) and coronal (C) gadolinium (Gd) T1-weighted MRI images. Postoperative MRI (D) shows no residual tumor.

In 2008, a 56-year-old woman presented with headaches and diplopia. The patient had a history of asthma and no significant family history. Computerized tomography (CT) and magnetic resonance imaging (MRI) revealed a pineal mass with heterogeneous enhancement without hydrocephalus (Figure 1).

**Figure 2:**
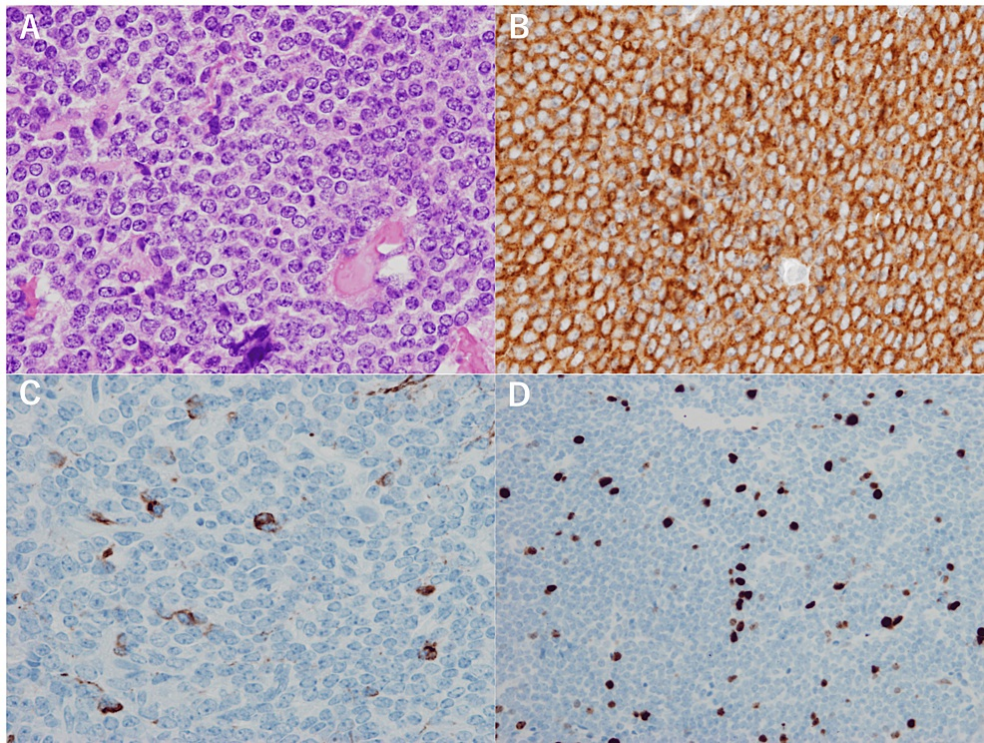
Pathological findings at the initial presentation A specimen stained with hematoxylin and eosin shows homogenous tumor cells with a high nuclear-cytoplasmic ratio and round nuclei (A). Immunohistochemical examinations show positive expression of synaptophysin (B) and neurofilament (C). The MIB-1 index is 10% (D). The magnification is ×200 in (A-C) and ×100 in (D).

Leptomeningeal dissemination was not identified on whole-spine MRI. Microsurgical gross total tumor resection (GTR) was performed. Immunohistochemical examination revealed focally positive expression of neurofilaments; the mitotic count was 5-10 per high-power field (hpf), and the MIB-1 index was 10% (Figure 2).

The pathological diagnosis was PPTID (grade 3). The MRI revealed no residual tumor after surgery. However, due to the grade 3 status, we administered adjuvant radiochemotherapy. She underwent whole brain radiation therapy (WBRT) at 30 Gy in 15 fractions and focal boost at 24 Gy in 12 fractions, followed by combination chemotherapy with cisplatin, nimustine, and vincristine. The patient was unable to continue chemotherapy for severe myelosuppression after the first course of treatment. A head MRI was performed every six months, and no recurrence findings were observed.

**Figure 3:**
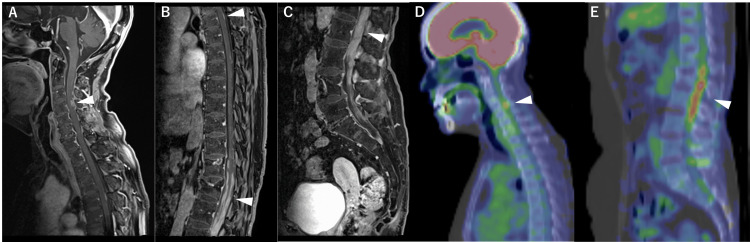
Images of spinal dissemination Whole spine Gd T1-weighted MRI shows enhanced mass lesions (arrowheads) at C3-4 (A), T4 (B), and cauda equina (C). FDG-PET shows accumulations in the same lesions (D, E).

In 2016, at the age of 64, she started to fall easily and complained of pain in her lower limbs and back. In 2019, 11 years after the surgery, she was unable to walk, and a whole-spine MRI performed by a primary doctor revealed multiple masses. She returned to our hospital, complaining of dysesthesia below the L1 dermatome and bilateral lower extremity weakness. Her Mini-Mental State Test (MMSE) score was 15, and her Hasegawa Dementia Scale-Revised (HDS-R) score was 11, indicating moderate cognitive disturbance as a late adverse effect of radiotherapy and chemotherapy. Whole-spine MRI in our hospital revealed multiple enhanced masses at C3-4, T4, and cauda equina. Fluorodeoxyglucose-positron emission tomography (FDG-PET) revealed accumulations of the same lesions. No recurrence was observed on head MRI and FDG-PET (Figure 3).

**Figure 4:**
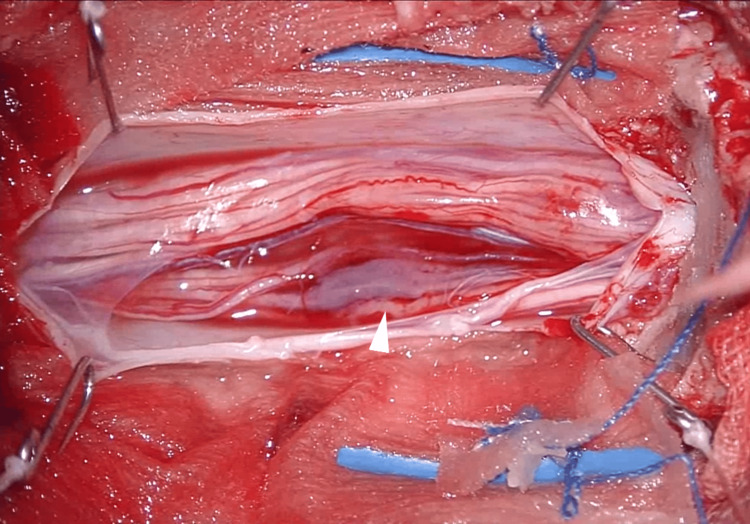
Photograph of the surgical field under a microscope Laminectomy and dural incision are performed. Tumor adherent to cauda equina (arrowhead).

A biopsy of the caudal portion was performed to determine the pathology of the tumor (Figure 4). 

**Figure 5:**
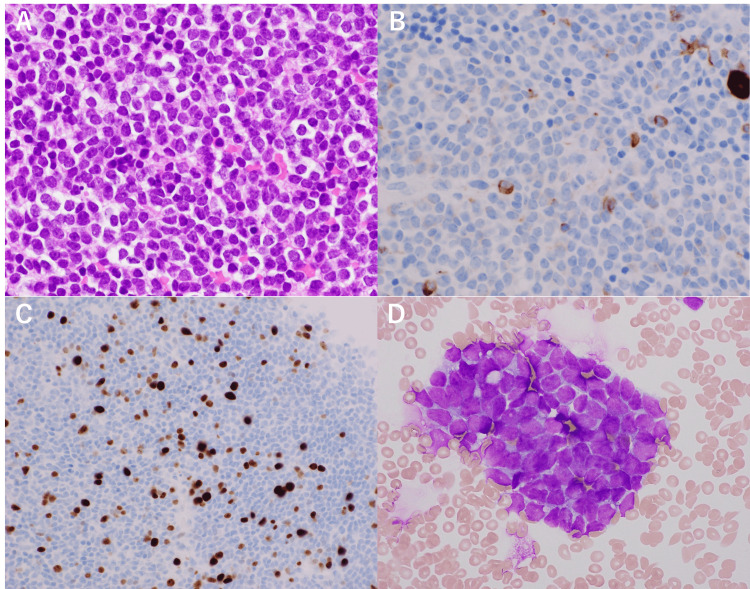
Pathological findings of spinal dissemination A specimen stained with hematoxylin and eosin is similar to that of the initial presentation (A). Neurofilament is focally positive as well (B). The MIB-1 index is 5-10% (C). Tumor cells are found in spinal fluid collected intraoperatively (D). The magnification is ×200 in (A, B), ×100 in (C), and ×600 in (D).

The histopathological findings were the same as those of the initial operation and the diagnosis of dissemination of PPTID (WHO grade 3) in the spine (Figure 5).

**Figure 6:**
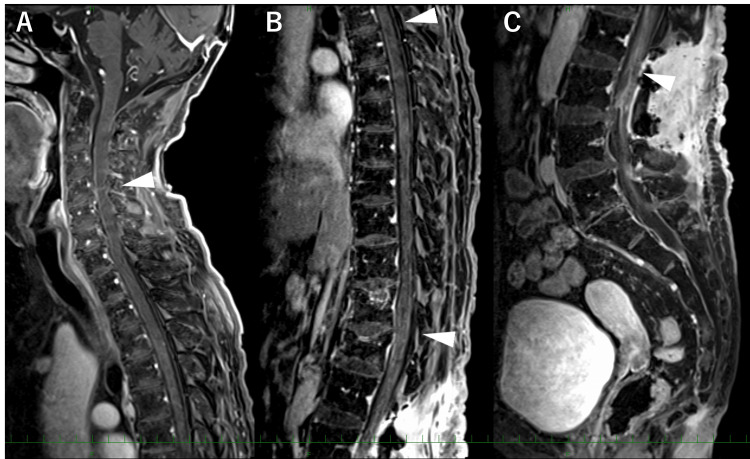
Images after treatment Whole spine Gd T1-weighted MRI shows tumor regression after radiation therapy.

She underwent ICE therapy (a combination of isofamide, cisplatin, and etoposide) before radiotherapy to evaluate the response to chemotherapy. A whole-spine MRI revealed no regression of the tumor after the first course of ICE therapy. She withdrew from chemotherapy and started whole spine radiotherapy at 36 Gy in 20 fractions, followed by a focal boost at 9 Gy in 5 fractions. Tumor regression was observed in whole-spine MRI after radiation therapy; moreover, her symptoms resolved (Figure 6).

An MRI performed in November 2023 showed no re-enlargement of spinal cord lesions.

## Discussion

PPTID is a rare tumor that includes WHO grades 2 and 3. The criteria for grade classification have not been established, and the WHO classification has not set any clearly defined criteria to distinguish between grades 2 and 3 [3]. However, each has a different prognosis; the five-year survival rate is 74% for WHO grade 2 and 39% for WHO grade 3 [6]. Therefore, it is clinically important to distinguish between WHO grades 2 and 3. Previous studies have comprehensively determined the grade based on histopathological and immunohistochemical examinations [7,8]. PPTID WHO grade 2 has <6 mitoses and is strongly positive for neurofilaments, whereas PPTID WHO grade 3 has >6 mitoses or <6 mitoses without strong immunostaining for neurofilaments [1]. Also, the presence of necrosis, mitotic rate, and immunohistochemical expression of neurofilament proteins are used to classify the grade [2]. The MIB-1 labeling index (MIB-1 LI) ranged from 5.2% ± 0.4% in PPTID WHO grade 2 to 11.2 ± 2% in PPTID WHO grade 3 [9]. In this case, histopathology showed typical findings of PPTID grade 3. Recently, genome sequencing has demonstrated that PPTID has a KBTBD4 mutation [10]. Yamashita et al. collected patients with PPTID and reported that 80% (20/25) of the patients harbored a KBTBD4 mutation [11]. This patient was included in the study and was found to harbor a KBTBD4 mutation. They reported that female sex (p = 0.018) and GTR (p < 0.01) were independent prognostic factors for progression-free survival (PFS), and female sex (p = 0.019) was associated with overall survival (OS). Further data accumulation based on molecular diagnosis is warranted to determine whether the KBTBD4 mutation is a prognostic factor.

This patient had no recurrence for over 10 years and survived for 4 years after recurrence, despite grade 3 recurrence. We considered two factors for long-term survival. The first is good local control with a combination of GTR and subsequent radiation therapy during the initial treatment. The second is to leave the option of spinal irradiation at the time of spinal dissemination. The optimal treatment for PPTID has not yet been determined. However, surgical excision is the standard of care, and previous studies have reported that GTR is a predictor of survival [12,13]. The role of radiotherapy has also been highlighted in previous studies because of the difficulty of surgery and its good radiosensitivity [4]. PPTID patients have the possibility of long-term survival; hence, there is a risk of late adverse effects. However, the tendency of PPTID for local recurrence as well as cerebrospinal dissemination is known [5]. Therefore, first of all, long-term follow-up of the spine and head is important. The extent of radiation therapy has not yet been determined. In this case, whole-spine radiotherapy with focal boost was performed at the time of spinal cord dissemination for the first time; therefore, delayed radiation myelopathy was avoided and may result in a long-term prognosis. The efficacy of chemotherapy remains controversial. There is no standard chemotherapy regimen for PPTID. Combination regimens, including platinum and etoposide, are often used [5]. We tried ICE therapy for spinal dissemination, but no regression was observed. Hence, the radiation option is more important.

The question of whether spinal cord irradiation should be performed as a primary treatment in patients without cerebrospinal dissemination is being discussed. Liu et al. compiled the literature on radiation therapy for PPTID [14]. They suggest the possibility of long-term control if cerebrospinal irradiation (CSI) is provided with positive spinal fluid and diffuse dissemination at initial presentation or as a salvage. Webb et al. reported clinical courses for patients with PPTID at their institution [15]. Five patients underwent GTR without recurrence, regardless of adjuvant radiation. One of five received CSI and had cognitive deficits, while two who received local radiation therapy showed no cognitive deficits. They hypothesized the possibility of achieving similar disease control and a more favorable toxicity profile with local radiation therapy than with CSI. However, it should be noted that most previous reports were retrospective and included a small number of patients.

## Conclusions

We report a case of long-term survival with PPTID WHO grade 3. No recurrence was observed over 10 years, owing to good local control with GTR and subsequent radiation to the brain. The spinal dissemination was resistant to chemotherapy and responded to whole spine radiation therapy, and no further recurrence occurred in four years. The importance of long-term follow-up of the spine and head is emphasized. In addition, performing only brain radiation when GTR is achieved in cases without cerebrospinal dissemination and withholding spinal radiation until spinal dissemination occurs may become a long-term treatment plan.
